# Active Surveillance Versus Immediate Surgery: Questionnaire Survey on the Current Treatment Strategy for Adult Patients with Low-Risk Papillary Thyroid Microcarcinoma in Japan

**DOI:** 10.1089/thy.2019.0211

**Published:** 2019-11-14

**Authors:** Iwao Sugitani, Yasuhiro Ito, Akira Miyauchi, Tsuneo Imai, Shinichi Suzuki

**Affiliations:** ^1^ Department of Endocrine Surgery, Nippon Medical School Graduate School of Medicine, Tokyo, Japan.; ^2^ Research Group for the Management of Papillary Thyroid Microcarcinoma, Japan Association of Endocrine Surgery & Japanese Society of Thyroid Surgery, Tokyo, Japan.; ^3^ Department of Surgery, Kuma Hospital, Kobe, Japan.; ^4^ Department of Surgery, National Hospital Organization, Higashinagoya National Hospital, Nagoya, Japan.; ^5^ Department of Thyroid and Endocrinology, Fukushima Medical University School of Medicine, Fukushima, Japan.

**Keywords:** questionnaire survey, papillary thyroid microcarcinoma, management, active surveillance, immediate surgery

## Abstract

***Background:*** Two Japanese prospective trials of active surveillance (AS) for adult patients with low-risk papillary thyroid carcinoma (PTC) ≤1 cm (cT1aN0M0 PTMC) have verified the safety of AS in oncological control and its superiority over immediate surgery with respect to unfavorable outcomes. Thus, AS has been accepted as an alternative to immediate surgery for asymptomatic papillary thyroid microcarcinomas (PTMCs). However, the real-world clinical approach for PTMC is unknown. Thus, this study aimed to investigate the current state of management of asymptomatic PTMCs in Japan.

***Methods:*** We conducted a questionnaire survey on the actual treatment patterns for adult patients with low-risk PTMCs. The subjects were member institutions of the Japan Association of Endocrine Surgery (JAES) or Japanese Society of Thyroid Surgery (JSTS), including the departments of surgery and head and neck surgery (HNS).

***Results:*** Responses were obtained from 134 institutes, where 72.4% of Japanese thyroid cancer cases operated by surgeons were treated. For suspicious tumors on ultrasound, 18 responders (13.4%) conducted cytological examination routinely, while 69 (51.5%) and 40 (27.8%) conducted it only for tumors >5 and >10 mm, respectively. After the diagnosis, 42 responders (31.3%) recommend AS, 35 (26.1%) recommend immediate surgery as the management, and 52 (38.8%) allowed patients to decide the treatment course. The present responders tended to recommend surgery for PTMCs that were located adjacent to the dorsal surface of the thyroid, were multiple, or measured almost 10 mm in size. At these institutions, 1176 patients with PTMC underwent surgery in 2017, accounting for 18.1% of surgeries for PTC. During the succeeding three months, 310 of 576 (53.8%) PTMC patients underwent AS. The treatment strategies did not differ between the departments (Surgery or HNS). The institutions that have six or more surgeons, that were located in metropolitan areas, or that were certified by JAES or JSTS performed AS more actively.

***Conclusion:*** More than 50% of low-risk PTMCs are on AS in Japan. However, the indication and recommendation for AS vary significantly between institutions. To improve the implementation of this management modality, physicians and patients should be further educated, and the sociomedical environment should be improved.

## Introduction

The incidence of thyroid carcinoma has rapidly increased recently in many developed countries (1–4) owing to the increase in the detection of small papillary thyroid carcinomas (PTCs), most of which are 10 mm or smaller and are called papillary thyroid microcarcinomas (PTMCs). The advances in and widespread use of imaging modalities including ultrasound and ultrasound-guided fine-needle aspiration cytology (FNAC) and the increased access to such imaging studies have led to the detection of low-risk PTMC (cT1aN0M0), which was previously mostly detected only on autopsy (5). Despite the increase in incidence, the mortality from thyroid carcinoma has remained stable (1–4), raising a question of whether surgical treatment of low-risk PTMCs is beneficial.

In Japan, a screening study of thyroid cancer among adult women using ultrasound revealed a 3.5% incidence of small PTC (6), which was >1000 times higher than the prevalence of clinical thyroid cancer in Japanese women being reported at that time (7). This prompted the two high-volume centers, Kuma Hospital (beginning in 1993) and Cancer Institute Hospital of Japanese Foundation for Cancer Research (beginning in 1995), to initiate a clinical trial of active surveillance (AS) of low-risk PTMC (i.e., PTMC without high-risk features such as clinical node or, although rare, distant metastasis, and symptoms of carcinoma extension such as recurrent laryngeal nerve paralysis and tracheal invasion). PTMCs attaching to the trachea or located in the course of the recurrent laryngeal nerve were regarded unsuitable for AS, although these features are not necessarily biologically aggressive. Long-term large-scale prospective studies from these two institutions showed favorable outcomes (8–11). AS of low-risk PTMC was then included in the guidelines developed by the Japan Association of Endocrine Surgery (JAES)/Japanese Society of Thyroid Surgery (JSTS) (12) and the American Thyroid Association (ATA) (13). Favorable results of AS from other countries such as the United States and Korea have also been reported (14–16).

However, even in Japan, it remains unclear how asymptomatic PTMCs are managed. Therefore, we performed this questionnaire survey for member institutions of JAES and/or JSTS to investigate the current state of the management of asymptomatic PTMCs in Japan.

## Patients and Methods

### Survey overview

A questionnaire survey entitled “Investigations on the actual management of adult papillary thyroid microcarcinoma” was sent via e-mail on August 7, 2018, to 1798 members of JAES and/or JSTS in 1177 medical institutes, which included the Department of Surgery and the Department of Head and Neck Surgery (HNS) or Otorhinolaryngology. Only one response was allowed from each medical center, but two responses were allowed if both the surgery and the head and neck departments manage and treat PTC in a single medical center. The response included the name of the institute and the name of the responder. It was sent back to the JAES office by e-mail. When we received two or more responses from the same department, we asked the responders to unify the reply and resubmit it. We requested the representative of each department to answer the basic policy of the institution instead of responders' personal opinions. The initial deadline was October 1, 2018. It was extended to November 1, 2018, and we sent a reminder mail to institutions that did not answer. The final date of collection of the questionnaire was November 14, 2018.

During the present questionnaire study, JAES and JSTS were unified to form a new society named the “Japan Association of Endocrine Surgery” on October 26, 2018. The members of the Japan Association of Endocrine Surgery are composed of endocrine surgeons (about 60%), head and neck surgeons (about 20%), urologist specializing in adrenal surgery (about 15%), and other medical professionals, including radiologists and pathologists, among others. JAES and JSTS have grant certification to endocrine surgeons with five year or more involvement in endocrine surgery training, a defined number of surgical experiences (e.g., 100 or more thyroid surgeries) and some research achievements. Institutions have been certified for the training of endocrine surgeons by JAES and JSTS when they have one or more full-time certified endocrine surgeon with a sufficient number of operations in the training curriculum. There are 190 certified institutions in Japan, and most academic centers and hospitals specializing in thyroid disease are certified.

This study was approved by the ethical committee of the Nippon Medical School on July 23, 2018 (No. 30-05-935) and by the ethical committees of JAES and JSTS on July 30, 2018.

### Questionnaire

The design and content of the questionnaire was discussed and determined by the members of the Research Group for the Management of Papillary Thyroid Microcarcinoma. The questionnaire includes three clinical questions regarding the management of nodules measuring ≤10 mm detected on ultrasound and suspected of malignancy but lacking the clinical findings of lymph node metastasis and significant extrathyroidal extension such as recurrent laryngeal nerve paralysis and tracheal invasion. In addition, there were two more questions focused on medical experience for each institution. All questions exclusively concerned the management of adult PTC patients.

Question 1. Select one of the following regarding the current indication of FNAC at your institute:

a. FNAC is performed for all nodules regardless of size.
b. FNAC is performed for nodules >5 mm, while nodules ≤5 mm are observed without FNAC.
c. FNAC is performed only for nodules >10 mm, while all nodules ≤10 mm are observed without FNAC.
d. The cutoff size for indication of FNAC is individually set.
e. Others.

Question 2. Select one of the following regarding the current fundamental explanation and treatment policy at your institute for the nodules cytologically diagnosed as low-risk PTMC:

a. Surgery is immediately recommended.
b. Two options, immediate surgery and AS, are presented to the patients, and they choose the strategy. However, physicians recommend surgery as the better choice.
c. Two options, immediate surgery and AS, are equally presented to the patients without indicating the physician's recommendation.
d. Two options, surgery and AS, are presented to the patients for their selection. Physicians recommend AS as the first-line management.
e. Others.

Question 3. Select the following regarding the current indications at your institute to actively recommend surgery for low-risk PTMCs. None or multiple answers were allowed.

a. Multiple PTMCs
b. Family history of differentiated thyroid cancer
c. Wish for conception
d. Age 60 years or older
e. Age 40 years or younger
f. Tumor size close to 10 mm
g. Tumor located near the dorsal capsule of the thyroid lobe
h. Others

Question 4. Provide the number of thyroid surgeries performed at your institute from January to December 2017.

Question 5. Provide the number of patients managed with surgery and AS for cT1aN0M0 PTMCs in your institute during a three-month period of your choice between January 2017 and September 2018.

### Thyroid cancer surgeries in the National Clinical Database

To estimate the coverage of the present survey on Japanese PTMC cases, we extracted data on the number of patients who underwent surgery for PTC from the National Clinical Database (NCD). The NCD is maintained by the Japan Surgical Society (JSS) and the members of JSS register all the surgeries they conducted into the database every year. All surgeons from JAES/JSTS are members of JSS.

### Statistical analysis

JMP^®^ 12.0.1 (2015 SAS Institute, Inc.) was adopted for statistical analysis. The chi-square test was used for analyzing the relationship between replies of questionnaire and the characteristics of responders (Tables 2–5). A *p*-value of <0.05 was considered significant.

## Results

### Questionnaire recovery rate and backgrounds of institutions

Of the 1177 institutions surveyed, 134 responded. Of the responders, 81 (60.4%) were the departments of surgery and 48 (35.8%) were the departments of HNS, and the remaining 5 were other departments. Furthermore, 95 (70.9%) of the responses were from institutions certified by JAES or JSTS (Table 1). Forty-five (33.6%) responders were located in the seven major metropolitan areas, including Sapporo district (Sapporo City), Sendai district (Sendai City), Kanto district (Tokyo, Yokohama City, Saitama City, Kawasaki City, Chiba City, and Sagamihara City), Chukyo district (Nagoya City), Kinki district (Kyoto City, Osaka City, Kobe City, and Sakai City), Hiroshima district (Hiroshima City), and Kitakyushu/Fukuoka district (Kitakyushu City and Fukuoka City). The number of thyroid surgeons varied from 0 to 6 or more. The response rate based on the number of institutions was low at 11.4%. However, according to the NCD database, of the 6971 thyroid cancer cases treated by JSS surgeons in 2017, 5046 (72.4%) were treated at the 81 responding Department of Surgery. Thus, coverage of the response based on the number of thyroid cancer cases was considerably high.

**Table 1.** Background of Responding Institutions

Department	
Surgery	81 (60.4%)
Head and Neck Surgery	48 (35.8%)
Others	5 (3.7%)
Institutions certified by JAES or JSTS	
Yes	95 (70.9%)
No	39 (29.1%)
Location	
Seven major metropolitan areas^a^	45 (33.6%)
Others	89 (66.4%)
Number of thyroid surgeons	
0	4 (3.0%)
1	18 (13.4%)
2	27 (20.1%)
3–5	68 (50.7%)
6 or more	17 (12.7%)

^a^ Sapporo district (Sapporo City), Sendai district (Sendai City), Kanto district (Tokyo, Yokohama City, Saitama City, Kawasaki City, Chiba City, and Sagamihara City), Chukyo district (Nagoya City), Kinki district (Kyoto City, Osaka City, Kobe City, and Sakai City), Hiroshima district (Hiroshima City), and Kitakyushu/Fukuoka district (Kitakyushu City and Fukuoka City).

JAES, Japan Association of Endocrine Surgery; JSTS, Japanese Society of Thyroid Surgery.

### Responses

The following were the responses to the survey questionnaire:

Question 1. The current indication of FNAC.

**FIG. 1.**
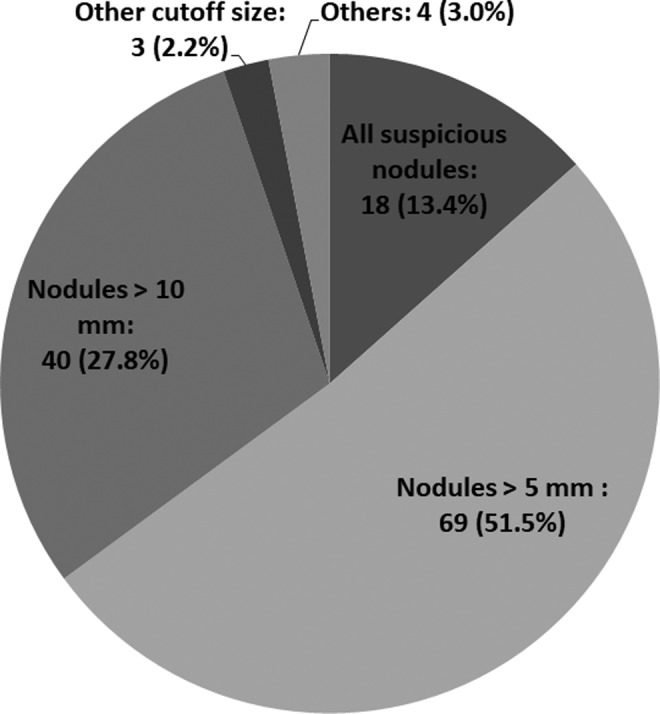
Indication of fine-needle aspiration cytology for thyroid nodules with suspicious ultrasound features.

As shown in Figure 1, 51.5% of the institutions performed FNAC for nodules >5 mm, 27.8% performed FNAC for nodules >10 mm, and 13.4% perform FNAC for nodules regardless of their sizes. Three responders set the cutoff size individually and the cutoff was 3 mm, 7 mm, and unspecified. Three institutions responded with variable comments.

Question 2. The current fundamental explanation and treatment policy for the nodules cytologically diagnosed as low-risk PTMC.

**FIG. 2.**
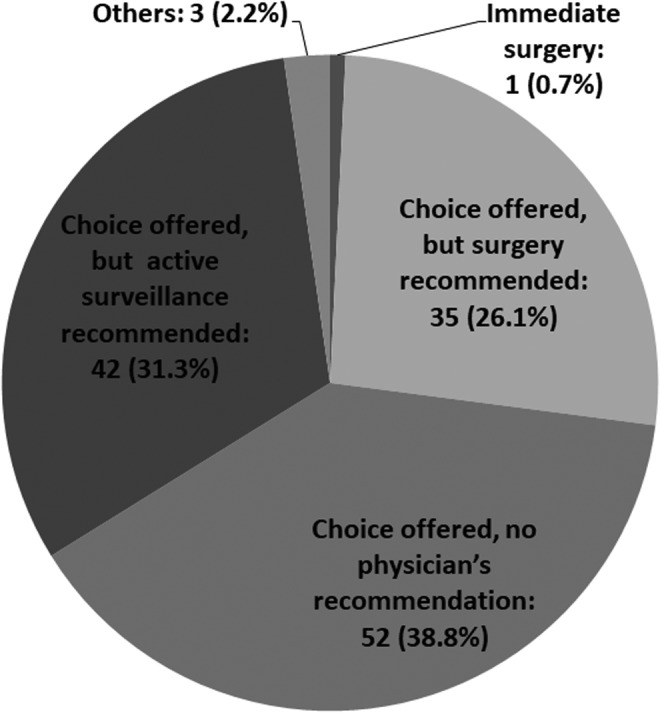
Current treatment policy for low-risk PTMC. PTMC, papillary thyroid microcarcinoma.

Most of the responding institutions present the patients with two options, AS and surgery. The frequency of physician discretion not affecting the patient's choice was highest at 38.8%, and 31.3% recommend AS as first-line management. However, 26.1% of the institutions still recommend surgery as first-line management. Only one responder recommended immediate surgery. “Others” was selected by three responders with variable comments. One responder did not answer this question (Fig. 2).

Question 3. The current conditions to actively recommend surgery for low-risk PTMCs.

**FIG. 3.**
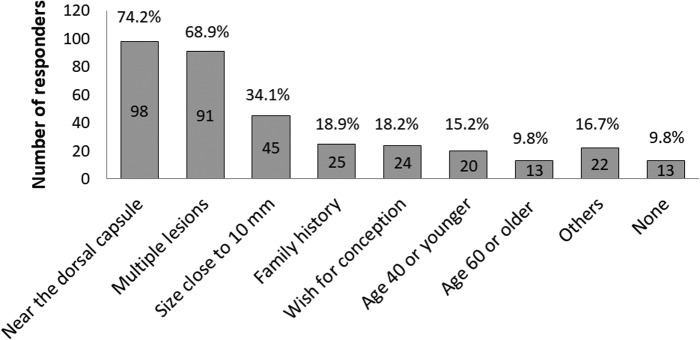
Current indications for recommending surgery in low-risk PTMC.

As shown in Figure 3, 119 institutions listed one or more conditions for actively recommending surgery for low-risk PTMC including a tumor located near the dorsal capsule of the thyroid lobe (74.2%), multiplicity (68.9%), tumor size close to 10 mm (34.1%), family history of differentiated thyroid carcinoma (18.9%), wish for conception (18.2%), age 40 years or younger (15.2%), and age 60 years or older (9.8%). Thirteen (9.8%) reported no specific conditions to recommend surgery.

Question 4. The number of thyroid surgeries performed from January to December 2017.

The total number of thyroid surgeries performed was 12,710. The number of initial surgeries for adult patients with PTC was 6493 (range: 0–750, average 48.8 ± 104.0). The number of cT1aN0M0 patients was 1176 (range: 0–216, average 8.8 ± 26.9). The number of cT1aN1aM0, cT1aN1bM0, and cT1a any N M1 patients was 139, 149, and 10, respectively. Thus, low-risk PTMC accounted for 18.1% of the 6493 adult patients with PTC who underwent thyroid surgery in 2017 among the 134 responders.

Question 5. The number of patients managed with surgery and AS for cT1aN0M0 PTMCs during a recent three-month period.

The total number of cT1aN0M0 patients treated at the 134 institutes was 576 (range: 0–143, average 4.4 ± 15.3). Immediate surgery was performed in 266 patients, and AS was selected in 310 patients (53.8%).

### Relationship between the characteristics of the institutions and the management of low-risk PTMC

The indication of FNAC was not related to the characteristics of the institutions, such as the type of department (Surgery vs. HNS), JAES or JSTS certification, location within the seven metropolitan areas or not, and the number of surgeons (Table 2). Moreover, the treatment policy and management did not differ according to the characteristics of the institutions (Table 3). However, in departments with 6 or more surgeons, patients were more frequently recommended AS (50.0%) as the first-line management strategy than others with fewer surgeons, although no statistical difference was identified.

**Table 2.** Relationship Between Indication of Fine-Needle Aspiration Cytology for Suspicious Thyroid Nodules and Characteristics of Responding Institutions

	*Indication of FNAC*			
	*All nodules*	*Nodules >5 mm*	*Nodules >10 mm*	p
Department				
Surgery	12 (15.6%)	43 (55.8%)	22 (28.6%)	0.72
Head and Neck Surgery	6 (13.3%)	23 (51.1%)	16 (35.6%)	
Certification from JAES or JSTS				
Yes	11 (12.5%)	50 (56.8%)	27 (30.1%)	0.62
No	7 (17.9%)	19 (48.7%)	13 (33.3%)	
Location				
Seven major metropolitan areas^a^	4 (9.8%)	21 (51.2%)	16 (39.0%)	0.32
Others	14 (16.5%)	48 (56.5%)	23 (27.1%)	
Number of thyroid surgeons				
1–2	4 (9.3%)	24 (55.8%)	15 (34.9%)	0.45
3–5	13 (19.7%)	35 (53.0%)	18 (27.3%)	
6 or more	1 (7.1%)	7 (50.0%)	6 (42.9%)	

^a^ Sapporo district (Sapporo City), Sendai district (Sendai City), Kanto district (Tokyo, Yokohama City, Saitama City, Kawasaki City, Chiba City and Sagamihara City), Chukyo district (Nagoya City), Kinki district (Kyoto City, Osaka City, Kobe City and Sakai City), Hiroshima district (Hiroshima City) and Kitakyushu/Fukuoka district (Kitakyushu City and Fukuoka City).

FNAC, fine-needle aspiration cytology.

**Table 3.** Relationships Between Informed Consent and Characteristics of Responding Institutions

	*Informed consent*			
	*Surgery recommended*	*No physician recommendation*	*Surveillance recommended*	p
Department				
Surgery	20 (26.0%)	33 (42.9%)	24 (31.1%)	0.94
Head and Neck Surgery	13 (27.1%)	19 (40.0%)	16 (33.3%)	
Certification from JAES or JSTS				
Yes	22 (23.7%)	41 (44.1%)	30 (32.3%)	0.26
No	13 (36.1%)	11 (30.6%)	12 (33.3%)	
Location				
Seven major metropolitan areas^a^	11 (25.6%)	17 (39.5%)	15 (34.9%)	0.92
Others	24 (27.9%)	35 (40.7%)	27 (31.4%)	
Number of thyroid surgeons				
1–2	12 (27.3%)	17 (38.6%)	15 (34.1%)	0.37
3–5	20 (30.3%)	29 (43.9%)	17 (25.8%)	
6 or more	2 (12.5%)	6 (37.5%)	8 (50.0%)	

^a^ Sapporo district (Sapporo City), Sendai district (Sendai City), Kanto district (Tokyo, Yokohama City, Saitama City, Kawasaki City, Chiba City, and Sagamihara City), Chukyo district (Nagoya City), Kinki district (Kyoto City, Osaka City, Kobe City, and Sakai City), Hiroshima district (Hiroshima City), and Kitakyushu/Fukuoka district (Kitakyushu City and Fukuoka City).

Table 4 shows the relationship between the characteristics of the departments and the incidence of low-risk PTMC accounting for the surgical cases of PTC. The incidence of low-risk PTMC in surgical cases was significantly higher in departments of surgery than that in HNS departments. Also, the incidence was significantly higher in institutions certified by JAES or JSTS, not located in the seven major metropolitan areas, and having a low number of surgeons.

**Table 4.** Relationship Between the Incidence of Surgical Cases of cT1aN0M0 Accounting for Adult Papillary Thyroid Carcinoma and Characteristics of the Responding Institutions

	*No. of surgical cases of adult PTC*	*No. of surgical cases of cT1aN0M0*	*%*	p
Department				
Surgery	5046	1005	19.9	<0.0001
Head and Neck Surgery	1447	171	11.8	
Certification from JAES or JSTS				
Yes	5777	1066	18.5	0.048
No	716	110	15.4	
Location				
Seven major metropolitan areas^a^	3867	624	16.1	<0.0001
Others	2626	552	21.0	
Number of thyroid surgeons				
1–2	741	167	22.5	0.0029
3–5	3471	633	18.2	
6 or more	2221	376	16.9	

^a^ Sapporo district (Sapporo City), Sendai district (Sendai City), Kanto district (Tokyo, Yokohama City, Saitama City, Kawasaki City, Chiba City, and Sagamihara City), Chukyo district (Nagoya City), Kinki district (Kyoto City, Osaka City, Kobe City, and Sakai City), Hiroshima district (Hiroshima City), and Kitakyushu/Fukuoka district (Kitakyushu City and Fukuoka City).

PTC, papillary thyroid carcinoma.

The relationship between the management of low-risk PTMC and the characteristics of the institutions is summarized in Table 5. The number of patients who underwent AS was significantly higher at institutions located in the seven major metropolitan areas, those with a high number of surgeons, and those certified by JAES or JSTS.

**Table 5.** Relationship Between the Management of cT1aN0M0 Papillary Thyroid Carcinoma and Characteristics of the Responding Institutions

	*Total number*	*No. of patients who underwent active surveillance*	*%*	p
Department				
Surgery	480	264	55.0	0.21
Head and Neck Surgery	93	44	47.3	
Certification from JAES or JSTS				
Yes	511	283	55.4	0.047
No	65	27	41.5	
Location				
Seven major metropolitan areas^a^	357	229	64.1	<0.0001
Others	219	81	37.0	
Number of thyroid surgeons				
1–2	76	31	40.8	<0.0001
3–5	213	85	40.0	
6 or more	287	194	67.6	

^a^ Sapporo district (Sapporo City), Sendai district (Sendai City), Kanto district (Tokyo, Yokohama City, Saitama City, Kawasaki City, Chiba City, and Sagamihara City), Chukyo district (Nagoya City), Kinki district (Kyoto City, Osaka City, Kobe City, and Sakai City), Hiroshima district (Hiroshima City), and Kitakyushu/Fukuoka district (Kitakyushu City and Fukuoka City).

### Responder comments

The primary comments from the responders are listed in Table 6. There were many valuable comments regarding AS. Regarding the diagnosis (A in Table 6), eight responders commented on the standardization of ultrasound diagnosis and its accuracy (A-1), and two commented on the discrepancy in the indication of FNAC between the ATA guidelines and the Japanese guidelines (A-2). Regarding the indication and contraindications of AS (B in Table 6), clear indications other than tumor size were requested (B-1). In particular, responders asked how to manage PTMCs with multiplicity and/or family history, high thyroglobulin levels and low thyrotropin levels, and high-grade malignancy on cytology (B-2, -3, and -4). Regarding implementing AS (C in Table 6), eight responders asked how long or until what age should patients undergo AS (C-1). Also, four responders asked about features of PTMC that lead to an indication for conversion surgery (C-4). Regarding education of patients and physicians (D in Table 6), 20 responders commented on the importance of forming a consensus and establishing management guidelines by our medical and surgical society and dissemination of guidelines to physicians, including primary care doctors, patients, and the general public (D-1). In this context, six responders commented on the difficulty in refusing surgery for patients who were diagnosed with PTMC on cytology, who were told to undergo surgery, and were then referred for surgery by primary care doctors (D-2). Concerning the improvement of the social medical environment (E in Table 6), nine responders emphasized the urgent need for staff recruitment, including ultrasound technicians and medical specialists, and education (E-1).

**Table 6.** Representative Comments from the Responders

		Number of comments
A. Regarding the diagnosis of nodules ≤10 mm suspected of PTC		
1	The standardization of ultrasound diagnosis and improving its accuracy are important, including the measurement of tumor size, evaluation of extrathyroidal extension, and lymph node metastases.	8
2	The indication of FNAC should be clarified. The discrepancy between the Japanese “Guidebook for ultrasound diagnosis of thyroid diseases” and the ATA guidelines 2015 limits accurate diagnosis and treatment.	2
B. Regarding the indications and contraindications of AS		
1	Clear presentation about indications other than tumor size is requested.	2
2	Clarify how to manage PTMCs with multiplicity and/or family history.	2
3	Clarify how to manage PTMCs with high serum thyroglobulin levels and low TSH levels.	2
4	Clarify how to manage when high-grade malignancy such as tall cell valiant is suspected on cytology.	1
C. Regarding implementing AS		
1	Define how long or until what age AS should be continued?	8
2	The appropriate frequency of surveillance is unknown.	6
3	Guidelines about examination, particularly evaluation of distant metastasis and its frequency are needed.	5
4	Features of PTMC that indicate a need to convert from AS to surgery should be clarified.	4
D. Regarding education for patients and physicians		
1	It is important to form a consensus, and JAES/JSTS should establish management guidelines and disseminate the information to physicians, primary care doctors, patients, and the general public.	20
2	It is difficult to refuse surgery for patients who were cytologically diagnosed with PTMC and were referred for surgery by primary care doctors.	6
3	Information should be carefully constructed to avoid misunderstanding such as that all PTCs are harmless and can be observed, and that none of the nodules measuring 10 mm or smaller requires close examination.	3
E. Suggestions for improvement of the sociomedical environment to implement AS		
1	Recruitment and education of staff, medical specialists, and ultrasound technicians are urgently needed.	9
2	Immediate surgery is more economically beneficial for hospital management, precluding implementation of AS. Compensation through an “active surveillance management fee” should be considered.	4
3	Countermeasures to avoid loss to follow-up is important.	2
F. Regarding future research agenda		
1	The establishment of a nationwide, long-term continuous case accumulation survey system for assessing outcomes of AS.	11
2	Further studies on PTMC with poor outcomes.	7
3	Methods for the early identification of PTMC progression (molecular markers etc.).	4
4	Studies on the patient perspective.	3
5	Significance of TSH suppression for patients who undergo AS.	2
6	Comparison of lifetime cost between AS and immediate surgery.	2

AS, active surveillance; ATA, American Thyroid Association; FNAC, fine-needle aspiration cytology; JAES, Japan Association of Endocrine Surgery; JSTS, Japanese Society of Thyroid Surgery; PTC, papillary thyroid carcinoma; PTMC, papillary thyroid microcarcinoma; TSH, thyrotropin.

In Japan, AS is less costly than immediate surgery (17). In relation to this, four responders suggested that AS be compensated through an “active surveillance management fee” in the Japanese Health Care Insurance System (E-2). There were also several suggestions regarding the future research agenda (F in Table 6). Eleven responders suggested the establishment of a nationwide, long-term continuous case accumulation survey system (F-1). Finally, three responders suggested studies addressing the patient perspective (F-4).

## Discussion

This is the first survey on the current management of low-risk PTMC in Japan. Although the recovery rate of this questionnaire was only 11.4% based on the number of responding institutes, JAES and JSTS have members who do not directly manage patients with thyroid disease, such as urologists, pathologists, radiologists, and gastrointestinal surgeons. According to the NCD, 6971 thyroid cancer cases were treated by JSS surgeons in 2017, and 5046 (72.4%) of them were treated at the 81 responding departments of surgery. Therefore, the response rate in the present survey covers the majority of institutes that treat thyroid carcinomas in Japan.

The ATA 2015 guidelines do not require FNAC for nodules ≤10 mm unless apparent signs of high-risk malignancy, such as recurrent laryngeal nerve paralysis and clinical node or distant metastasis are present (13). In Japan, FNAC for nodules >5 mm with highly suspicious ultrasound features such as invasiveness has been recommended by the Japan Association of Breast & Thyroid Sonology since 2016 (18). In the present survey, 51.5% of the institutions performed FNAC for nodules >5 mm following the guidebook, while 27.8% performed FNAC for nodules >10 mm following the ATA recommendation. Also, 13.4% perform FNAC for nodules regardless of their sizes. It remains debatable whether small thyroid nodules with suspicious ultrasound features should be evaluated with FNAC. The ATA guidelines do not recommend FNAC for nodules ≤10 mm to avoid unnecessary thyroid surgery for these tumors and to preclude patient concerns of having an untreated carcinoma. However, some PTMCs progress during AS. Informing patients regarding the diagnosis of PTMC can encourage them to undergo regular checkups. The present questionnaire revealed that, in Japan, the majority of institutions perform FNAC for suspicious nodules ≤10 mm and propose AS as the management choice of PTMC.

Most of the responding institutions present the patients with two options, AS and surgery, and share the decision-making with their patients. Among them, the incidence of physician discretion not affecting the patients' choice was highest, at 38.8%, and 31.3% recommend AS as first-line management. However, 26.1% of the institutions still recommend surgery as the first-line management despite several studies reporting the safety of AS for oncological control and its superiority over immediate surgery with respect to adverse outcomes (19). This is possibly due to a lack in experience in AS among these attending physicians. As shown in Table 3, 50% of institutions with 6 or more surgeons recommended AS as first-line management, and the rate of recommending AS was higher than in those with smaller numbers of surgeons.

In this survey, 119 institutions listed one or more conditions for actively recommending surgery for low-risk PTMC including a tumor located near the capsule on the dorsal side on imaging studies, multiplicity, tumor size close to 10 mm, family history of differentiated thyroid carcinoma, wish for conception, age 40 years or younger, and age 60 years or older. Ito *et al.* previously demonstrated that the angle formed by the tumor surface and the tracheal cartilage, and the presence of a normal rim between the tumor and the capsule, is important for evaluating tumor invasion to the trachea and the recurrent laryngeal nerve, respectively. They showed that not all low-risk PTMC located in the dorsal side should be operated (20). Multiplicity, size, and family history were not regarded as a contraindication for AS because they did not predict PTMC growth on multivariate analysis (9–11). Also, one study showed that only a subset of PTMC enlarges during pregnancy and, if enlarged, surgery after delivery remains safe (21). Although low-risk PTMC in young patients is more likely to progress (9–11), Miyauchi *et al.* estimated that more than 50% of patients who are in their 20s at presentation and ∼75% of the patients in their 30s will not require surgery in their lifetime (22). Furthermore, older patients have a lower probability of needing surgery (22). Miyauchi *et al.* conducted a kinetic analysis on tumor volume of PTMCs during AS and reported that only 3% of the tumors showed rapid growth, 22% showed slow growth, 57% showed stable disease, and 17% showed a decrease in their tumor volume over time (23). To further promote AS and avoid unnecessary surgery, both attending physicians and patients should be educated about AS.

In the present responder institutions, the proportion of low-risk PTMC among PTCs surgically treated in 2017 was 18.1% (1176 of 6493). In these institutions, more than half (53.8%) of low-risk PTMC patients underwent AS. However, the present survey shows that the incidence of AS varies among institutions.

The frequency of surgery for low-risk PTMC was higher in departments of surgery than in HNS departments. Furthermore, the frequency was also higher in certified institutions. However, the frequency of AS was also higher in these institutions. Furthermore, in institutions located in the seven major metropolitan areas and those with a large number of surgeons, the incidence of low-risk PTMC accounting for surgically treated PTC was significantly lower, and the incidence of low-risk PTMC patients who underwent AS was significantly higher. These findings suggest that low-risk PTMC patients tend to visit certified institutions, and AS is more actively performed in high-volume centers in metropolitan areas. This is possibly because patients living in urban areas have more opportunities to be educated about AS, and institutions with a large number of staff can afford to perform AS more easily.

Responders also commented on many important issues in the management of PTMC, as shown in Table 6. These comments identified several barriers to a more widespread implementation of AS that will be useful for stakeholders interested in increasing its acceptance more broadly. First, several responders emphasized that the education of ultrasonographers and medical specialists is an urgent need. For appropriate management of low-risk PTMC, a medical team comprising a skilled sonographer with a high-quality ultrasound machine and an experienced endocrinologist or thyroid surgeon is essential, as previously stated by Brito *et al.* (24). Second, they requested the standardization of diagnosis and accuracy of ultrasound and the establishment of guidelines for AS that should be disseminated not only to attending physicians but also to primary care doctors and patients. These strategies are considered crucial to broaden the implementation of AS for PTMC. Improving the knowledge of primary care doctors is helpful for a multidisciplinary management of PTMC patients. Third, a nationwide, long-term continuous case accumulation survey for information sharing is desirable, which could be useful to collect a spectrum of information. Finally, regarding the patient perspective, a joint study by the Dartmouth Institute in the United States and Kuma Hospital reported that 83% of the patients on AS at Kuma Hospital replied that choosing AS was the best decision for them personally, although 37% of them continued to worry about their cancer from time to time (25).

This study has some limitations. First, although the design and content of the questionnaire was deliberately discussed and determined by the members of the research group, the survey instrument was not a validated tool and we did not conduct a pilot test to clarify the internal validity. Second, we requested that a representative of each department answer the basic policy of the institution. Thus, the responses do not represent all personal views of Japanese physicians. In addition, the results could be biased because of the nonanonymous structure of the survey. Finally, this survey covers members of JAES and JSTS involved in the management and treatment of thyroid carcinomas. Therefore, the responding physicians should be more knowledgeable than general doctors, possibly causing a bias of this study. Furthermore, the respondents were mostly high-volume centers. Thus, the findings may not reflect the current clinical practice at small-volume institutes.

In conclusion, this survey shows a high rate of AS for PTMCs in Japan. However, marked differences in the indication and recommendation for AS among institutions were also observed. To increase the use of AS for PTMC, physicians, primary care doctors, patients, and their families should be educated about AS, and the sociomedical environment should also be improved.
